# Evaluation of Cynomolgus Macaque *(Macaca fascicularis)* Endogenous Retrovirus Expression Following Simian Immunodeficiency Virus Infection

**DOI:** 10.1371/journal.pone.0040158

**Published:** 2012-06-29

**Authors:** Angie K. Marsh, David O. Willer, Olena Skokovets, Oluwadamilola H. Iwajomo, Jacqueline K. Chan, Kelly S. MacDonald

**Affiliations:** 1 Department of Immunology, University of Toronto, Toronto, Ontario, Canada; 2 Department of Medicine, University of Toronto, Toronto, Ontario, Canada; 3 Department of Microbiology, Mount Sinai Hospital, Toronto, Ontario, Canada; Commissariat a l’Energie Atomique(cea), France

## Abstract

Human endogenous retrovirus type K (HERV-K) transcripts are upregulated in the plasma of HIV-infected individuals and have been considered as targets for an HIV vaccine. We evaluated cynomolgus macaque endogenous retrovirus (CyERV) mRNA expression by RT-qPCR in PBMCs isolated from a cohort of animals previously utilized in a live attenuated SIV vaccine trial. CyERV env transcript levels decreased following vaccination (control and vaccine groups) and CyERV env and gag mRNA expression was decreased following acute SIV-infection, whereas during chronic SIV infection, CyERV transcript levels were indistinguishable from baseline. Reduced susceptibility to initial SIV infection, as measured by the number of SIV challenges required for infection, was associated with increased CyERV transcript levels in PBMCs. *In vitro* analysis revealed that SIV infection of purified CD4^+^ T-cells did not alter CyERV gene expression. This study represents the first evaluation of ERV expression in cynomolgus macaques following SIV infection, in an effort to assess the utility of cynomolgus macaques as an animal model to evaluate ERVs as a target for an HIV/SIV vaccine. This non-human primate model system does not recapitulate what has been observed to date in the plasma of HIV-infected humans suggesting that further investigation at the cellular level is required to elucidate the impact of HIV/SIV infection on endogenous retrovirus expression.

## Introduction

Despite major advances in antiretroviral therapy, the HIV/AIDS epidemic continues to spread. Researchers are striving to prevent HIV acquisition through vaccination, however the very nature of HIV poses significant challenges to the development of a successful HIV vaccine. HIV rapidly mutates and recombines under immune selection pressure leading to extreme antigenic diversity and hypervariability (reviewed in [1], [2]). Conventional vaccine approaches have been largely ineffective against this degree of viral diversity. One unique approach under investigation is to examine HIV-infected cells to determine if the virus causes changes to cellular proteins that may act as surrogate markers for HIV infection and thus represent novel vaccine targets. Recently, human endogenous retroviruses (HERVs) have emerged as potential alternative cellular targets for this type of HIV vaccine strategy [3], [4].

HERVs are remnants of ancient retroviral infections, fixed in our genome and transmitted vertically [5]. Integration of HERVs has occurred over millions of years and over time they have acquired point mutations rendering them unable to produce infectious virions [6], [7]. The youngest retrovirus in the HERV family is the *betaretrovirus* HERV-K (HML-2), which was the most recent HERV to integrate into the genome and thus has the highest degree of transcriptional activity [8]. Furthermore, of the 31 defined HERV families [9], the sequence of HERV-K is the most homologous to HIV and is the most widely examined HERV in the field of HIV [10]. The relationship between HERV-K and HIV emerged from initial reports demonstrating that antibodies against HERV-K (HML-2) were found in 70% of HIV-positive patients compared with only 3% of HIV-naïve individuals [6], [11]. Furthermore, it has been shown that HERV-K RNA titers are elevated in the plasma of HIV-infected individuals in comparison with seronegative individuals [3], [12], [13], [14]. Most intriguing for vaccine design is a recent study demonstrating elevated and functional T-cell responses in HIV-positive patients against cellular protein targets derived from HERV-K [3]. Given that HERVs are encoded in the germ-line, it is hypothesized that they are not subjected to the same degree of cytotoxic T lymphocyte (CTL) immune pressure and subsequent immune escape as HIV antigens. In addition, plasma HERV-K RNA titers are inversely correlated with the degree of HIV suppression following highly active antiretroviral therapy (HAART) suggesting that HERV-K may be a predictor of HIV replication [12], although the mechanism by which HIV impacts HERV-K transcriptional processes is not well understood.

Many studies have examined HERV-K expression in the plasma of HIV-infected patients; however, little work has been done to determine if HERV-K is activated at the cellular level. It is known that peripheral blood mononuclear cells (PBMCs) express HERV-K gag and pol RNA [15]. The limitations of many human *in vivo* studies are that these findings are derived from independent cross-sectional samples. A longitudinal study using a non-human primate model will allow for endogenous retrovirus (ERV) expression to be assessed pre- and post-SIV infection from dependent samples, providing a precise measurement of changes in ERV expression as a result of viral infection.

Cynomolgus macaques (*Macaca fascicularis*) are a widely utilized non-human primate model for preclinical biomedical research [16]. This species of macaques is susceptible to pathogenic simian immunodeficiency virus (SIV) infection and ultimately succumbs to simian AIDS (SAIDS). Although the duration of SIV pathogenesis and progression to SAIDS is shorter in macaques than in humans [17], cynomolgus macaques closely recapitulate HIV disease progression and thus are a practical model to assess endogenous retrovirus expression. In this study, we evaluated cynomolgus macaque endogenous retrovirus (CyERV) expression in PBMCs following SIV infection. Here we show the characterization of CyERV genes and the effect of vaccination and SIV infection on CyERV expression levels in PBMCs.

## Results

### Isolation and Genetic Characterization of CyERV Genes

**Figure 1:**
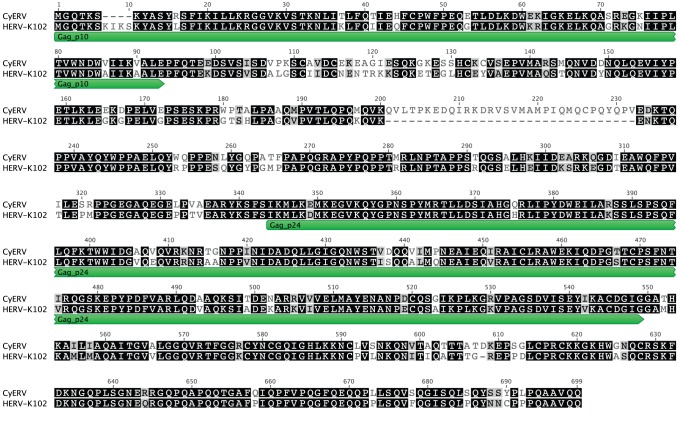
Amino acid alignment of CyERV gag with HERV-K102 gag. The protein sequence of CyERV gag (GenBank: JN985533) was aligned with HERV-K102 (GenBank: P63130) using ClustalW and shaded using Geneious Pro 5.4.6 [31]. HERV-K102 is annotated with the matrix protein (Gag_p10) and the nucleocapsid protein (Gag_p24). Black shading indicates identical residues found at both sites amongst the aligned proteins and grey shading indicates similar amino acid residues.

Full-length CyERV envelope and gag genes were PCR-amplified and sequenced to examine the diversity between CyERVs as well as to determine if cynomolgus macaque endogenous retroviruses encode intact open reading frames (ORFs). With respect to nucleotide identity, the isolated CyERV clones showed a high degree intra-animal homology for env (avg. 98% identity) and gag (avg. 94% identity), and inter-animal homology for env (91% identity; n = 5) and gag (81% identity; n = 4). A BLAST search of the CyERV env and gag sequences revealed homology to HERV-K, while homology to other known HERV families was not observed. The amino acid length (avg. 695 aa) and molecular weight (avg. 75 kDa) of the CyERV genes were comparable to HERV-K genes (env 694 aa, 78 kDa and gag 666 aa, 74 kDa) [18]. The majority of the CyERV env and gag amino acid sequences contained multiple stop codons likely as a result of acquired point mutations. In this regard, the CyERV env clones were of two distinct genotypes. Of the 10 CyERV env clones (2 clones/animal) isolated, half represented genotype 1 (19 stop codons; 696 aa) and half represented genotype 2 (29 stop codons; 691 aa), while each genotype shared 99.4% nucleotide identity between clones. From our analysis, these stop codons are likely the result of point mutations with genotype 1 resulting from 7 indels and 4 substitutions while genotype 2 was derived from 9 indels and 5 substitutions. To evaluate the amino acid identity between CyERV env and HERV-K env (GenBank: CAA76886), we resolved the indels/substitutions in the CyERV sequence and determined that genotypes 1 and 2 share 86.5% and 87.9% amino acid identity, respectively, with HERV-K env. The CyERV gag isolates contained an average of 27 stop codons (range: 0–47), however one isolate encoded a complete CyERV gag ORF (GenBank: JN985533) with 695 amino acids and a predicted molecular weight of 77.7 kDa. This complete CyERV gag clone shares 77.4% identity with HERV-K102 gag (GenBank: P63130) at the amino acid level (Fig. 1). The discovery of an intact CyERV gag ORF supports previous documentation that cynomolgus macaques may have retained the capacity to produce full-length CyERV gag proteins [19].

### Impact of Vaccination on CyERV Transcript Expression Levels in PBMCs

**Figure 2:**
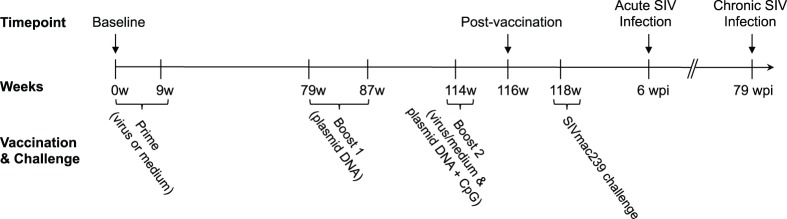
Vaccination schedule. Baseline PBMC samples were obtained prior to any vaccinations (week 0). The animals were primed at weeks 0 and 9 with viral constructs (Δ5-CMV, Δ6-CCI) or medium only (control group). Animals were boosted with a DNA plasmid (Δ5-CMV, Δ6-CCI or control) at weeks 79 and 87. A second boost comprised of both virus and plasmid accompanied by a CpG adjuvant was given at week 114. Post-vaccination PBMCs were isolated at week 116 and intrarectal SIVmac239 challenge was initiated at week 118. PBMCs were isolated during acute SIV infection (mean 6 weeks post-infection (wpi); range: 4–10 wpi) and during chronic SIV infection (mean 79 wpi; range 25–98 wpi) [20].

**Figure 3:**
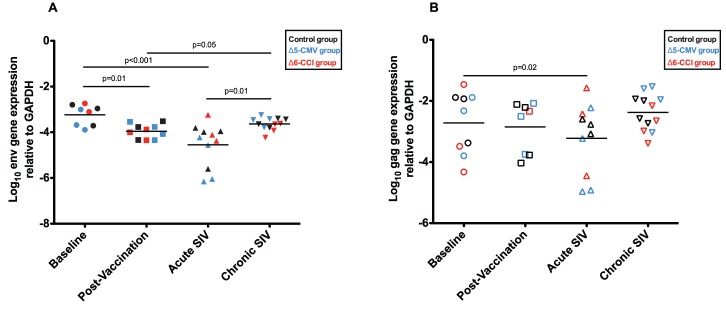
CyERV gene expression in peripheral blood mononuclear cells. CyERV envelope and gag RNA was isolated from PBMCs and quantified using RT-qPCR. Log transformed CyERV envelope (A) and gag (B) gene expression levels relative to GAPDH were compared across all timepoints (baseline, post-vaccination, acute and chronic SIV infection). Post-vaccination PBMCs are comprised of samples from both week 114 and 116. Vaccine groups include controls, Δ5-CMV group, and Δ6-CCI group. Paired-samples t-tests (2-tailed) were performed to determine statistical significance, bars represent the mean.

The SIV vaccine trial was comprised of two vaccine groups (Δ5-CMV and Δ6-CCI) and one control group (mock-immunized), and followed a prime-boost-boost immunization regimen. The animals in the vaccine groups were immunized with highly attenuated SIVmac239 viral constructs, as described in the Materials/Methods and Figure 2 [20]. To assess the impact of vaccination on CyERV gene expression levels, we extracted RNA from PBMCs isolated at baseline and post-vaccination timepoints to quantify CyERV env and gag gene expression by RT-qPCR. The post-vaccination timepoint is derived from samples taken at either week 114 or 116. No significant differences were observed between the two sampling weeks and as such these post-vaccination samples were grouped. There were no observed differences in CyERV env and gag expression between the three vaccine groups (controls, Δ5-CMV, and Δ6-CCI). In comparison with baseline expression (log_10_ −3.30), CyERV env transcript levels were significantly lower following vaccination (log_10_ −4.03; n = 6; P = 0.01) (Fig. 3A), with a less pronounced decrease in CyERV gag expression between baseline (log_10_ −2.53) and post-vaccination (log_10_ −3.04; n = 6; P = 0.33) (Fig. 3B).

### Downregulation of CyERV Transcript Levels Following SIV Infection in PBMCs

Following vaccination, the animals were challenged weekly with a multi-low-dose SIVmac239 intrarectal challenge regimen until productive plasma infection was detected by RT-qPCR [20]. We examined CyERV env and gag gene expression at two timepoints representing acute [avg. 6 weeks post-infection (wpi); range: 4–10 wpi] and chronic (avg. 79 wpi; range: 25–98 wpi) SIV infection (Fig. 2). CyERV gene expression was significantly decreased during acute SIV infection and returned to baseline levels during chronic SIV infection (Fig. 3). At the acute timepoint, the mean CyERV env expression level (log_10_ −4.74) was lower than baseline (log_10_ −3.24; n = 8; P<0.001) (Fig. 3A). CyERV env transcript expression increased during chronic SIV infection compared with post-vaccination (log_10_ −3.65 versus log_10_ −3.96; n = 10; P = 0.05) and acute SIV infection (log_10_ −3.61 versus log_10_ −4.55; n = 11; P = 0.01). Mean CyERV gag expression decreased following acute SIV infection (log_10_ −3.40) compared with baseline levels (log_10_ −2.40; n = 7, P = 0.02) and was comparable with baseline during chronic SIV infection (Fig. 3B). Of note, when the above data was normalized with respect to CD4^+^ T-cell counts, CyERV gene expression patterns were analogous (data not shown).

### Susceptibility to SIV Infection was Associated with CyERV Expression Levels in PBMCs

**Figure 4:**
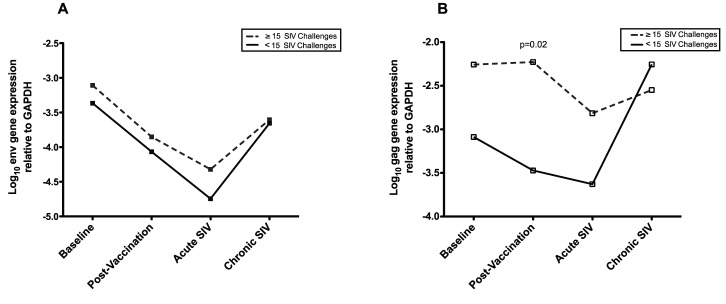
CyERV gene expression is associated with decreased susceptibility to SIV infection. A comparison between animals that required ≥15 challenges with SIV (range: 15–25, mean: 19.2) to become infected versus animals that required <15 SIV challenges (range: 2–9, mean: 4.9). A) Animals that were less susceptible (≥15 SIV challenges) had higher CyERV envelope expression levels at all timepoints. There was no statistically significant difference between the two groups. B) Animals that were less susceptible (≥15 SIV challenges) had higher CyERV gag expression levels at baseline, post-vaccination and acute SIV infection. At the post-vaccination timepoint, there was a statistically significant difference between the two groups (p = 0.02). Independent-samples t-tests (2-tailed) were performed to determine the statistical significance between groups. Data points represent the mean CyERV values for each group.

Susceptibility to SIV infection was determined based on the number of SIVmac239 challenges required to infect the cynomolgus macaques during the challenge phase of the trial [20]. Consistent with the grouping assigned by Willer et al. [20], the groups were based on the number of SIV challenges (<15 vs. ≥15 challenges) to infection, irrespective of vaccination or control group. CyERV env gene expression levels in the animals that required ≥15 SIV challenges (n = 5) to establish infection were elevated across all timepoints compared with the animals that were infected with SIV in <15 challenges (n = 7) (Fig. 4A), although there was no statistically significant difference between the two groups. Likewise, CyERV gag expression in the animals that required ≥15 SIV challenges to establish infection was increased across all timepoints except during chronic SIV infection where gag expression levels were slightly lower compared with animals that were infected with SIV in <15 challenges (Fig. 4B). CyERV gag transcript levels were significantly higher at the post-vaccination timepoint in the animals that were less susceptible to SIV infection (log_10_ −2.23) compared with those that were more susceptible (log_10_ −3.47; P = 0.02).

### CyERV Gene Expression Associates with CD4^+^ T-cell Counts and SIV Viral Load

**Figure 5:**
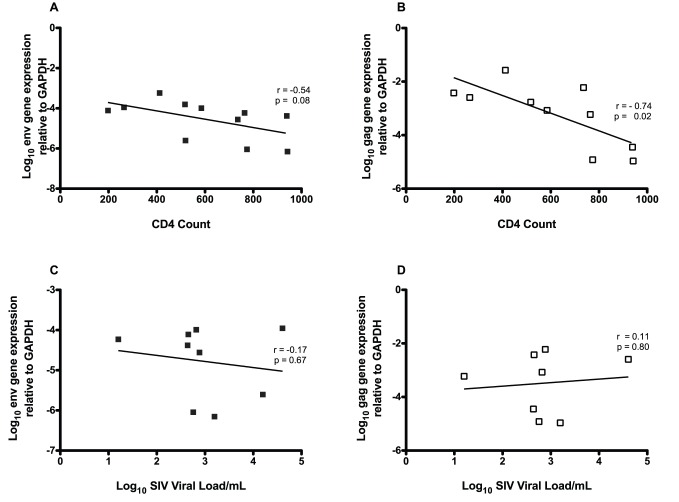
CyERV gene expression negatively associated with CD4^+^ T-cell counts and SIV viral load. During acute SIV infection, CyERV envelope (n = 11) and gag (n = 10) gene expression levels negatively correlated with CD4^+^ T-cell counts (CD4^+^ T-cells/ul of PBMCs) (A-B) and negatively associated with acute SIV viral load for CyERV envelope (n = 9) (C). CyERV gag (n = 8) expression showed a slight trend towards a positive association with acute SIV viral load (D). Bivariate correlations were performed using Pearson’s correlation coefficient (r), statistical significance values (p) are shown.

Given that acute SIV infection had the most significant impact on CyERV expression levels, we examined the relationship between CyERV expression and clinical markers of disease status using CD4^+^ T-cell counts (CD4^+^ T-cells/µl of PBMCs) and SIV viral load (log_10_ viral load/mL), previously determined during the vaccination study [20]. CyERV gene expression negatively correlated with CD4^+^ T-cell counts during acute SIV infection for env (r = −0.54; n = 11; P = 0.08) and gag (r = −0.74; n = 10; P = 0.02) (Fig. 5A-B). Additional correlations were performed using the percent CD4^+^ T-cells of total lymphocytes and the results were comparable to the absolute counts (data not shown). With respect to SIV viral load, CyERV env and gag gene expression did not significantly correlate with acute SIV viral load. CyERV Env expression (r = −0.17; n = 9; P = 0.67) showed a trend towards a negative association (Fig. 5C) in comparison with gag expression (r = 0.11; n = 8; P = 0.80), which displayed a trend towards a positive association (Fig. 5D). A similar analysis of CyERV expression was conducted during chronic SIV infection with no significant correlations observed with respect to either CD4^+^ T-cell counts or SIV viral load (data not shown).

### CyERV Transcript Expression in *in vitro* SIV-infected CD4^+^ T-cells

**Figure 6:**
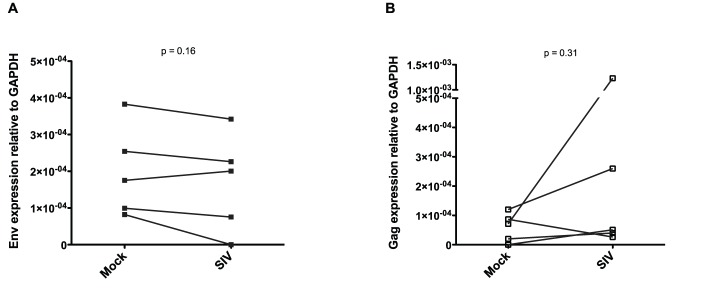
CyERV gene expression levels in CD4^+^ T-cells following *in vitro* SIV infection. CyERV gene expression was quantitated by RT-qPCR using RNA isolated from mock- and SIV-infected CD4^+^ T-cells. CyERV envelope (A) and gag (B) gene expression levels relative to GAPDH are compared between *in vitro* mock- and SIV-infected CD4^+^ T-cells. Paired-samples t-tests (2-tailed) were performed to determine statistical significance.

To examine CyERV gene expression in a single population of cells, CD4^+^ T-cells were enriched from cynomolgus macaque PBMCs and mock- or SIV-infected (SIVmac239) by magnetofection [21]. Total RNA was isolated from the mock- or SIV-infected cells to quantify CyERV env and gag expression by RT-qPCR. Although, hypothetically, all cell types have the potential to produce CyERVs, we specifically examined CD4^+^ T-cells since they are the cell type that is preferentially infected by SIV. CyERV transcript levels were examined in mock- and SIV-infected CD4^+^ T-cells to evaluate the effect of SIV infection on CyERV gene expression. CyERV env expression was not significantly altered in SIV-infected CD4^+^ T-cells compared with the mock-infected CD4^+^ T-cells (n = 5; P = 0.16) (Fig. 6A). Eighty percent (4/5) of the animals showed an increase in CyERV gag expression following SIV infection; however, no statistically significant difference was observed between mock- and SIV-infected CD4^+^ T-cells (n = 5; P = 0.31) (Fig. 6B). No correlation was observed between CyERV expression and the number of SIV-infected CD4^+^ T-cells (data not shown).

## Discussion

It is unclear how many CyERV proviruses have been incorporated into the cynomolgus macaque genome and whether they remain intact. The rhesus macaque (*Macaca mulatta)* genome contains nineteen complete proviruses of rhesus macaque endogenous retrovirus-K (RhERV-K) [22]; however, a similar analysis has yet to be performed in the two recently sequenced cynomolgus macaque genomes [23], [24]. Given that these Old World monkeys (OWMs) evolved from a common ancestor after the integration of HERV-K [25], [26], we would expect cynomolgus macaques to harbor a similar number of CyERV-K proviruses in their genome. Furthermore, after the divergence of hominoids and OWMs 25 million years ago, there have been at least 2750 OWM-specific ERV sequences identified and it is possible that these insertions may be more active [26]. A study examining the CyERV families and the number of proviruses present in the cynomolgus macaque genome would aid in elucidating the full CyERV complement and direct future CyERV-based SIV/HIV vaccine approaches. It is currently unclear if SIV selectively alters CyERV gene expression in certain families or if the phenomenon is universal. Our documentation of a full-length CyERV gag ORF (Fig. 1) indicates that cynomolgus macaques have retained the capacity to produce CyERV gag proteins, as previously described in a study examining gag sequences in OWMs [19]. Although we sequenced a number of CyERV env clones we were unable to isolate an intact ORF, supporting previous observations by Mayer et al. [19]; however, we cannot rule out the potential for full-length ORFs to be present. Our analysis was directed at characterizing CyERV env and gag, therefore it is possible that other intact CyERV ORFs (such as pol) are present in the cynomolgus macaque genome.

In the current study, CyERV expression levels were evaluated in PBMCs from a cohort of cynomolgus macaques utilized in a live attenuated SIV vaccine trial. As a protracted immunization was employed [20], we examined its impact on CyERV gene expression. Our results indicated that following the vaccination protocol there was a significant decrease in CyERV env expression levels and a marginal decrease in CyERV gag expression (Fig. 3). This was consistent between vaccinees and controls suggesting that perhaps a vaccine component(s) delivered to all groups (such as the DNA plasmid backbone) may have been responsible for the alterations in CyERV gene expression. In a recent study examining CyERV expression in the brains of cynomolgus macaques infected with bovine spongiform encephalopathy (BSE), the authors suggested that the observed decrease in macaque ERV-K (HML-2) gag RNA and protein expression may have been attributed, at least partially, to the inoculation procedure [27]. Ideally, to assess the impact of SIV infection on CyERV gene expression, a cohort of naïve animals that have not previously been vaccinated would be the preferred model. Of note, a comparison of CyERV gene expression levels with respect to the use of CpG as an adjuvant showed no effect on CyERV gene expression levels (data not shown). As such, it is possible that innate immune responses, although not mediated through CpG, may be playing a role in downregulating CyERV gene expression.

During acute SIV infection, CyERV env and gag gene expression levels were the lowest in comparison with all other timepoints (Fig. 3). If innate immunity is involved, maximal increase in innate immune activation during acute SIV infection could be contributing to the decrease in CyERV transcript levels. Preliminary data from our group examining the effects of Varicella Zoster Virus (VZV) infection on CyERV transcript expression showed similar results with decreased CyERV env and gag expression levels during acute VZV infection and a return to baseline levels during chronic VZV infection (unpublished data), further suggesting that the strong innate immune response during acute infection may be involved in decreasing CyERV gene expression.

Our observations that demonstrated disparate expression levels with respect to SIV susceptibility may be explained by a concept examined by Garrison et al. [3] in which they suggest that HERV-K-specific T-cells may cross-react with HIV-specific epitopes. In a separate study, they showed HERV-K-specific T-cells might be involved in the control of HIV during chronic infection [4]. In our study, animals that required a greater number of challenges with SIV to establish infection had higher CyERV env and gag transcript levels prior to SIV challenge and during acute SIV infection (Fig. 4). Thus, the positive association between CyERV expression levels and reduced susceptibility to SIV infection might imply that CyERV-specific cytotoxic CD8^+^ T-cells recognize SIV-specific epitopes thereby killing SIV-infected cells and contributing to the prevention of SIV infection. However, once SIV infection occurs our results show a trend towards a negative association between CyERV env expression levels and SIV viral load (Fig. 5C). This challenges the hypothesis that CyERV gene expression increases in SIV-infected cells. Furthermore, a negative correlation was observed between CyERV env and gag expression and CD4^+^ T-cell counts during acute SIV infection (Fig. 5A-B); whereas, during chronic SIV infection there was a trend towards a positive association between CyERV gene expression and CD4^+^ T-cell counts. Considering that CD4^+^ T-cells are a major target for SIV infection, we would expect to see the most robust effect of SIV infection on CyERV transcript levels in this subset of cells.

We examined *in vitro* SIV-infected cynomolgus macaque CD4^+^ T-cells to evaluate whether CyERV expressions was modulated by SIV infection. SIV infection had no impact on CyERV env expression; however, four out of five animals showed increased CyERV gag expression in the SIV-infected CD4^+^ T-cells (Fig. 6). Recently, Lefebvre et al. [28] used next-generation sequencing to examine cellular transcription of HERVs in an HIV-infected T-cell line and showed an insignificant increase in HERV-K transcription in the HIV-infected cells. Although the methodologies differed and their analysis was not specific to any one particular HERV protein, these similar results would suggest that intracellular endogenous retrovirus transcriptional events do not parallel the degree of upregulation observed by others examining HERV-K expression in HIV-infected plasma samples. Although plasma samples from this study were not available, matched sets of plasma and PBMCs would be useful in delineating any differences in HERV expression between the two sources.

Our findings from the evaluation of CyERV expression in PBMCs isolated from SIV-infected cynomolgus macaques were not consistent with what has been observed for HERV-K expression in the plasma of HIV-infected humans. During acute SIV infection, CyERV transcript levels were decreased and negatively associated with CD4^+^ T-cell counts. Future examination of CyERV expression in plasma and tissue samples would assist in further explaining whether these findings are unique to macaques or if this is a cellular phenomenon that has yet to be fully investigated in humans. Based on the results from this non-human primate study, an investigation into the direct effects innate immune activation and SIV infection on CyERV expression will be required before CyERVs can be evaluated as SIV vaccine targets.

## Materials and Methods

### Ethics Statement

The samples in this study are historical samples derived from a previous SIV vaccine trial [20] and no further samples were collected for the current study. All animal work for the original SIV vaccine trial was for research purposes and was approved in accordance with the Health Canada Institutional Animal Care Committee (protocol #2010–001), which met the ethical, scientific and social responsibility criteria set out by the Canadian Council on Animal Care (http://www.ccac.ca/Documents/Standards/Guidelines/Protocol_Review.pdf). For the SIV vaccine trial study, adult male colony-bred cynomolgus macaques (*Macaca fascicularis*) of Philippine origin were housed at the Animal Resources Division at the St. Frederick Banting Research Center (Ottawa, Canada). The veterinary staff monitored the animals daily for food intake, stool consistency and general welfare. The animals were anesthetized with ketamine (10 mg/kg) during all inoculations, viral challenges and sampling procedures. Any abnormal observation found during regular clinical evaluations was brought to the attention of veterinary staff. In situations of multiple systemic consequences, coupled with complete anorexia for more than three days resulting in weight loss of more than twenty percent, euthanasia was elected while the animal was under palliative care/feeding. The decision to euthanize the animal was always to ensure the animal would not suffer, and at no time was death considered an acceptable endpoint. Animals had large single cages exceeding the minimum requirements with areas of both privacy and visual social interaction with other animals. Animals were given a daily comprehensive program of environmental enrichment to prevent abnormal behavior and minimize stress. On alternate days, animals were given access to a large exercise area.

### Animals and Vaccine Constructs

All samples in this study are derived from a previous SIV vaccine trial [20], in which twelve adult male cynomolgus macaques of Philippine origin were randomly assigned into two experimental groups and one control group (4 animals in each group). Two highly attenuated vaccine constructs (Δ5-CMV and Δ6-CCI), derived from SIVmac239 [29], [30], were employed in the SIV vaccine trial. The vaccination schedule followed a prime-boost-boost regimen in which the animals were primed with viral constructs (Δ5-CMV or Δ6-CCI) or medium only (control animals), and subsequently boosted with plasmid DNA (Δ5-CMV, Δ6-CCI or control). For the second boost, the animals were given their respective viral and plasmid constructs (Δ5-CMV or Δ6-CCI), while the controls received medium alone and control plasmid DNA. All animals received a B-class CpG oligodeoxynucleotides (ODN) adjuvant with their viral construct and/or plasmid DNA during the second boost [20]. SIV inoculations were repeated weekly until a detectable SIV infection was established. Peripheral blood mononuclear cells (PBMCs) were isolated using routine methods [20] at various stages throughout the trial and stored at −150°C. Samples from the following timepoints were used for the current study: baseline (pre-vaccination), post-vaccination (2–4 weeks prior to challenge), acute SIV infection (mean 6 weeks post-infection (wpi); range 4–10 wpi) and chronic SIV infection (mean 79 wpi; range 25–98 wpi) (Fig. 2).

### Isolation of Cynomolgus Macaque Endogenous Retrovirus (CyERV) Genes

CyERV envelope and gag were isolated from PBMCs of five cynomolgus macaques (C09-001M, C09-002M, C09-005M, C09-008M, C09-009M) using a DNeasy Blood & Tissue Kit (Qiagen). CyERV env and gag genes were PCR amplified using published primer sequences based on HERV-K10 [19], forward primer (T7envFOR) 5′-ATGAACCCATCAGAGATGCA-3′ and reverse primer (envREV) 5′-AACAGAATCTCAAGGCAGAAGA-3′ for env; and forward primer (T7gagFOR) 5′-ATGGGGCAAACTAAAAGT-3′ and reverse primer (gagREV) 5′-CAGGCAGTGGGCCATATAC-3′ for gag. The PCR conditions were as follows: 95**°**C for 7 mins, 30 cycles of 95**°**C for 30 s, 52**°**C for 30 s, 72**°**C for 50 s and one cycle of 72**°**C for 7 mins. PCR amplicons were gel-purified with GENECLEAN II (MP Biomedicals), cloned into the pCR-Blunt II-TOPO vector (Invitrogen) and transformed with chemically competent cells (Invitrogen). The full-length gene inserts were confirmed by Sanger sequencing. Sequences were aligned by ClustalW alignment and shaded using Geneious Pro 5.4.6 [31].

### CD4^+^ T-cell Enrichment and *in vitro* SIV Infection

CD4^+^ T-cells were enriched from cynomolgus macaque (n = 5) PBMCs using a custom EasySep negative selection kit (StemCell Technologies). Enriched CD4^+^ T-cells were resuspended in R15–100 buffer (RPMI 1640 plus 15% FBS, L-glutamine, antibiotic/antimycotic) supplemented with 100 IU/ml rIL-2 and 5 µg/ml concanavalin A and cultured *in vitro* for 3–6 days. The CD4^+^ T-cells were either mock- or SIV-infected (SIVmac239) by magnetofection [21] for 48 hrs and stained with SIVmac p27 antibody (NIH AIDS Research and Reference Reagents Program) to evaluate SIV infection by FACS. Total RNA was isolated from the cell pellets.

### RNA Isolation

PBMCs were thawed at 37**°**C, washed with DMEM (Sigma-Aldrich) supplemented with 10% FBS (Wisent Bioproducts), 100 U/ml penicillin, 100 µg/ml streptomycin (Sigma-Aldrich), and subsequently washed with 1X Dulbecco’s phosphate buffered saline (Invitrogen). Total RNA was purified from the cell pellets using RNeasy Plus Mini kit (Qiagen) and eluted in 30 µl of RNase-free water, as per manufacturer’s protocol. The RNA was treated with 2 U TURBO DNase (Ambion) to remove any contaminating genomic DNA.

### Standard Curve Plasmid Cloning

A control plasmid for quantitative polymerase chain reaction (qPCR) was generated by incorporating the amplicons of CyERV env and gag, in conjunction with a reference gene, cynomolgus macaque glyceraldehyde 3-phosphate dehydrogenase (GAPDH). Restriction sites were incorporated by PCR to facilitate cloning of the genes into pMECA cloning vector (GenBank: AF017063). CyERV env primers were designed in regions conserved between the two clones of CyERV env, to amplify a 166 bp amplicon, FP 5′- ACGTGCGGCCGCTACCTGGCCCCACAGATGAC-3′ (NotI underlined) and RP 5′-ACGTCCATGGTACTTCTACCAACCAATTTTG-3′ (NcoI underlined). CyERV gag primers were designed in conserved regions between the two clones of CyERV gag, to amplify a 301 bp amplicon, FP 5′- ACGTGGATCCGGGCCTGGGAGAAAATCCAAG-3′ (BamHI underlined) and RP 5′-ACGTAGATCTTGCATAGCTCCTCCAATTCCATC-3′ (Bgl II underlined). The GAPDH primers were designed from a known *Macaca fascicularis* mRNA partial coding sequence (GenBank: DQ464111). PCR primers (FP 5′- ACTGGCATGCTGACCTGCCGTCTGGAAAA-3′ [SphI underlined] and RP 5′-ACGTGCTAGCCTCCGACGCCTGCTTCA-3′ [NheI underlined]) were designed to amplify an 80 bp region of GAPDH from cynomolgus macaque PBMCs. All gene fragments were cloned into pMECA (CyERV env; CyERV gag; GAPDH) and confirmed by Sanger sequencing. Serial dilutions of the plasmid were prepared to achieve a linear range of 8 logs (5 to 5^7^ copies/µl) and single use aliquots were stored at −80**°**C.

### Reverse Transcription-quantitative Polymerase Chain Reaction (RT-qPCR)

CyERV mRNA transcript levels were assessed by RT-qPCR. Total RNA (avg. 324 ng) was reverse transcribed to complementary DNA (cDNA) using SuperScript III First-Strand Synthesis SuperMix (Invitrogen) as per manufacturer’s protocol. Reverse-transcription negative controls and no template controls were included for each sample. All samples were loaded as technical triplicates. Thermal cycling conditions were: 95**°**C for 10 mins, 40 cycles of 95**°**C for 15 s, 60**°**C for 1 min followed by a dissociation stage of 95**°**C for 15 s, 60**°**C for 15 s and 95**°**C for 15 s. The primer sequences and amplicon sizes of each gene are listed in Table 1. Representative qPCR amplicons were analyzed by gel electrophoresis on a 2% agarose gel to confirm the size and purity of the products. In addition, select qPCR products were sequenced to confirm the transcripts. The RT-qPCR assay consistently demonstrated high efficiency and linearity for each gene (Table 1). The standard curve was used to enumerate CyERV mRNA expression. Sequence Detection System v2.4 (Applied Biosystems) was used for gene expression analysis with CyERV expression levels being standardized to 1 µg of RNA, normalized to the GAPDH reference gene and log transformed. Values represent the mean of the three technical replicates. Threshold cycle values or quantitative values that were deemed outliers based on Grubbs’ test (Z≥1.15) were excluded.

**Table 1:** **RT-qPCR Primers.**

Gene	Primer Sequence	Amplicon	Slope	Correlation
GAPDH	*F -* TGACCTGCCGTCTGGAAAA	68bp	−3.38	1.00
	*R* - CTCCGACGCCTGCTTCA			
CyERV Envelope	*F* - TACCTGGCCCCACAGATGAC	88bp	−3.41	1.00
	*R -* GGAGGATAATGATACCCAGTGGAA			
CyERV Gag	*F* - GGGCCTGGGAGAAAATCCAAG	289bp	−3.55	0.99
	*R* - TGCATAGCTCCTCCAATTCCATC			

The slope and correlation represent the mean value.

### Statistical Analysis

Oneway ANOVA tests were performed to compare the mean differences between the vaccine groups (Δ5-CMV, Δ6-CCI, control). Paired-samples t-tests (2-tailed) were performed to determine the statistical significance across various timepoints (dependent samples). For inter-group comparisons, independent-samples t-tests (2-tailed) were applied to determine the statistical significance. Bivariate correlations were performed using Pearson’s correlation coefficient (2-tailed). Statistical analysis was completed using SPSS for Macintosh (Rel. 19.0.0. 2010. SPSS Inc.).

## References

[1] Johnson WE, Desrosiers RC (2002). Viral persistence: HIV's strategies of immune system evasion.. Annual review of medicine.

[2] Goulder PJ, Watkins DI (2004). HIV and SIV CTL escape: implications for vaccine design.. Nature reviews Immunology.

[3] Garrison KE, Jones RB, Meiklejohn DA, Anwar N, Ndhlovu LC (2007). T cell responses to human endogenous retroviruses in HIV-1 infection.. PLoS pathogens.

[4] SenGupta D, Tandon R, Vieira RG, Ndhlovu LC, Lown-Hecht R (2011). Strong human endogenous retrovirus-specific T cell responses are associated with control of HIV-1 in chronic infection.. Journal of virology.

[5] Vogt PK, Coffin JM, Hughes, S.H, Varmus, H.E (1997). Historical introduction to the general properties of retroviruses..

[6] Lower R, Lower J, Kurth R (1996). The viruses in all of us: characteristics and biological significance of human endogenous retrovirus sequences.. Proceedings of the National Academy of Sciences of the United States of America.

[7] Nelson PN, Carnegie PR, Martin J, Davari Ejtehadi H, Hooley P (2003). Demystified. Human endogenous retroviruses.. Molecular pathology : MP.

[8] Kurth R, Bannert N (2010). Beneficial and detrimental effects of human endogenous retroviruses.. International journal of cancer Journal international du cancer.

[9] Katzourakis A, Tristem M, Sverdlov ED (2005). Phylogeny of human endogenous and exogenous retroviruses..

[10] van der Kuyl AC (2012). HIV infection and HERV expression: a review.. Retrovirology.

[11] Denner J, Phelps RC, Lower J, Lower R, Kurth R (1995). Antibody response of pregnant women, tumor and AIDS patients against the human endogenous retrovirus HERV-K.. Journal of Cancer Research and Clinical Oncology 121.

[12] Contreras-Galindo R, Almodovar-Camacho S, Gonzalez-Ramirez S, Lorenzo E, Yamamura Y (2007). Comparative longitudinal studies of HERV-K and HIV-1 RNA titers in HIV-1-infected patients receiving successful versus unsuccessful highly active antiretroviral therapy.. AIDS research and human retroviruses.

[13] Contreras-Galindo R, Gonzalez M, Almodovar-Camacho S, Gonzalez-Ramirez S, Lorenzo E (2006). A new Real-Time-RT-PCR for quantitation of human endogenous retroviruses type K (HERV-K) RNA load in plasma samples: increased HERV-K RNA titers in HIV-1 patients with HAART non-suppressive regimens.. Journal of virological methods.

[14] Contreras-Galindo R, Kaplan MH, Markovitz DM, Lorenzo E, Yamamura Y (2006). Detection of HERV-K(HML-2) viral RNA in plasma of HIV type 1-infected individuals.. AIDS research and human retroviruses.

[15] Medstrand P, Lindeskog M, Blomberg J (1992). Expression of human endogenous retroviral sequences in peripheral blood mononuclear cells of healthy individuals.. The Journal of general virology.

[16] O'Connor DH (2006). Chinese rhesus and cynomolgus macaques in HIV vaccine and pathogenesis research.. Future virology.

[17] Hu SL (2005). Non-human primate models for AIDS vaccine research.. Current drug targets Infectious disorders.

[18] Tonjes RR, Czauderna F, Kurth R (1999). Genome-wide screening, cloning, chromosomal assignment, and expression of full-length human endogenous retrovirus type K. Journal of virology.

[19] Mayer J, Meese E, Mueller-Lantzsch N (1998). Human endogenous retrovirus K homologous sequences and their coding capacity in Old World primates.. Journal of virology.

[20] Willer DO, Guan Y, Luscher MA, Li B, Pilon R (2010). Multi-low-dose mucosal simian immunodeficiency virus SIVmac239 challenge of cynomolgus macaques immunized with "hyperattenuated" SIV constructs.. Journal of virology.

[21] Sacha JB, Watkins DI (2010). Synchronous infection of SIV and HIV in vitro for virology, immunology and vaccine-related studies.. Nature protocols.

[22] Romano CM, de Melo FL, Corsini MA, Holmes EC, Zanotto PM (2007). Demographic histories of ERV-K in humans, chimpanzees and rhesus monkeys.. PloS one.

[23] Ebeling M, Kung E, See A, Broger C, Steiner G (2011). Genome-based analysis of the nonhuman primate Macaca fascicularis as a model for drug safety assessment.. Genome research.

[24] Yan G, Zhang G, Fang X, Zhang Y, Li C (2011). Genome sequencing and comparison of two nonhuman primate animal models, the cynomolgus and Chinese rhesus macaques.. Nature biotechnology.

[25] Steinhuber S, Brack M, Hunsmann G, Schwelberger H, Dierich MP (1995). Distribution of human endogenous retrovirus HERV-K genomes in humans and different primates.. Human genetics.

[26] Han K, Konkel MK, Xing J, Wang H, Lee J (2007). Mobile DNA in Old World monkeys: a glimpse through the rhesus macaque genome.. Science.

[27] Greenwood AD, Vincendeau M, Schmadicke AC, Montag J, Seifarth W (2011). Bovine spongiform encephalopathy infection alters endogenous retrovirus expression in distinct brain regions of cynomolgus macaques (Macaca fascicularis).. Molecular neurodegeneration.

[28] Lefebvre G, Desfarges S, Uyttebroeck F, Munoz M, Beerenwinkel N (2011). Analysis of HIV-1 expression level and sense of transcription by high-throughput sequencing of the infected cell.. Journal of virology.

[29] Guan Y, Whitney JB, Diallo K, Wainberg MA (2000). Leader sequences downstream of the primer binding site are important for efficient replication of simian immunodeficiency virus.. Journal of virology.

[30] Kestler H, Kodama T, Ringler D, Marthas M, Pedersen N (1990). Induction of AIDS in rhesus monkeys by molecularly cloned simian immunodeficiency virus.. Science.

[31] Drummond AJ, Ashton B, Buxton S, Cheung M, Cooper A (2011). Geneious v5.4..

